# *Aabrm1*-mediated melanin synthesis is essential to growth and development, stress adaption, and pathogenicity in *Alternaria alternata*

**DOI:** 10.3389/fmicb.2023.1327765

**Published:** 2024-01-11

**Authors:** Rong Li, Yongcai Li, Wenyi Xu, Wenjuan Liu, Xiaobin Xu, Yang Bi, Dov Prusky

**Affiliations:** ^1^College of Food Science and Engineering, Gansu Agricultural University, Lanzhou, China; ^2^Department of Postharvest Science of Fresh Produce, Agricultural Research Organization, Bet Dagan, Israel

**Keywords:** *Alternaria alternata*, melanin, brm1, infection, oxidative stress, virulence

## Abstract

Scytalone dehydratase (brm1) is one of the key enzymes in 1, 8-dihydroxynaphthalene (DHN) melanin synthesis, which mediates melanin biosythesis and regulates cell biological process of plant fungi, but its function in *Alternaria alternata*, the causal agent of pear black spot, is unclear. Brm1 in *A. alternata* was cloned, identified, and named as *Aabrm1*. An *Aabrm1*-deletion mutant was generated and revealed that the deletion of *Aabrm1* leads to a significant decrease in melanin production and forms orange colony smooth spores. In addition, the deletion of *Aabrm1* gene impaired infection structure information and penetration. The external stress resistance of Δ*Aabrm1* was significantly weakened, and, in particular, it is very sensitive to oxidative stress, and the contents of H_2_O_2_ and O_2_^.-^ in Δ*Aabrm1* were significantly increased. Virulence of Δ*Aabrm1* was reduced in non-wound-inoculated pear leaves but not changed in wound-inoculated pear fruit. These results indicated that *Aabrm1-*mediated melanin synthesis plays an important role in the pathogenicity of *A. alternata.*

## 1 Introduction

*Alternaria alternata*, as a familiar plant pathogenic fungus, can invade fruits during pre-harvest and post-harvest storage or transport by various pathways (Meena et al., 2017). Since *A. alternata* can grow at temperatures as low as −2°C, it is relatively difficult to control, especially when post-harvest fruits and vegetables are stored at low temperatures (Misaghi et al., 1977). *Alternaria alternata* can infect the host via three main pathways as follows: (1) directly penetrates the cuticular layer of the fruit epidermis, (2) the natural openings (lenticel and stomatal invasion), and (3) the wound (mechanical and insect injuries; Prusky, 1996). As a typical latent infection disease, the spore of *A. alternata* first adheres to the surface of pear fruit and then germinates and differentiates infection structures induced by surface physicochemical cues, finally activating multiple pathogenic factors and successfully penetrating fruits (Tang et al., 2017). Cell wall-degrading enzymes, melanin, toxins, and exopolysaccharides are the main pathogenic factors in plant fungi, which can destroy host cells or cause their physiological and metabolic dysfunction (Gao et al., 2020). Melanin, as a secondary metabolite of filamentous fungi, plays an important role in external stress adaption and pathogenicity (Chumley and Valent, 1990; Kawamura et al., 1997). However, the regulatory role of melanin and its accumulation under specific conditions on the development and pathogenicity of plant fungi, especially *A. alternata,* need to be further comprehensively elucidated.

Melanin is a polymeric compound resulting from the oxidation of polyhydroxyphenol and indole (Nosanchuk and Casadevall, 2006). According to the different intermediate metabolites, melanin is divided into 1, 8-dihydroxynaphthalene (DHN), 3, 4-dihydroxyphenylalanine (DOPA), γ-glutaminyl- 3, 4- dihydroxybenzene (GDBH), and catechol (Butler et al., 2001). DHN melanin is frequently accumulated in spores, appressoria, and aerial hyphae of plant pathogenic fungi during vegetative growth (Butler et al., 2001; Gao et al., 2022). The fungal DHN melanin biosynthetic pathway is usually based on acetate and involves a variety of enzymes including polyketide synthase (PKS), hydroxynaphthalene reductase (THN), scytalone dehydratase (SCD), α-hydrolases (Ayg), laccase (Lac), and a melanin synthesis transcription factor (CmrA) (Bell and Wheeler, 1986; Fujii et al., 2004; Wang Y. M. et al., 2018), and the genes encoding these enzymes are usually organized within gene clusters and can be expressed coordinately through the activation of transcription factors, which are also located within the gene clusters (Gao et al., 2022).

As a key gene in melanin synthesis, the *brm1* gene participates in the two-step reaction, including the dehydration of scytalone to trihydroxynaphthalene and vermelone to dihydroxynaphthalene. The brm1 gene has been reported in many fungi, such as *Botrytis cinerea* (Chen et al., 2021), *A. alternata* (Gao et al., 2022), *Sclerotinia sclerotiorum* (Liang et al., 2018), and it plays a critical role in melanin synthesis, growth and development, stress resistance, and pathogenicity. In *A. alternata*, deletion of the *brm1* gene reduced the production of altertoxin (ATX) (Gao et al., 2022). In *S. sclerotiorum*, loss of this gene affected growth and development and decreased UV irradiation resistance; however, there is no effect on pathogenicity (Liang et al., 2018). Deletion of *bcscd1* (*brm1* homologous gene) in *B. cinerea* decreased sporulation and sporogenesis germination of sclerotia (Chen et al., 2021). To further illustrate the function of *brm1* in *A. alternata*, the causal agent of pear black spot, *brm1,* in *A. alternata* was cloned in this study, and the regulatory role of the *Aabrm1* gene on growth, infection structure formation, oxidative stress, and virulence of *A. alternata* was studied using a targeted gene knockout technique; the results will be conducive to in-depth understanding of the function of melanin in fungi and developing new strategies to control post-harvest disease.

## 2 Materials and methods

### 2.1 Strains and plant material

After isolating the wild-type (WT) strain *A. alternata* JT-03 from pear fruit (*Pyrus bretschneideri*), it was identified and stored in our laboratory. All strains were preserved in an −80°C ultra-low temperature freezer and cultured in potato dextrose agar (PDA) plates at 28°C in the darkness. Configuration of spore suspensions (1 × 10^6^ spores ml − ^1^) follows the method of Tang et al. (2017). The vectors pCHPH and N-pCNEO were stored at −80°C refrigeration for later use. The test pear fruits (*Pyrus bretschneideri*) and pear leaves were provided by Tiaoshan Farm (Jingtai County, Gansu Province, China).

### 2.2 Targeted gene cloning and bioinformatics analysis

Samples of DNA and RNA were extracted using the method proposed Li et al. (2022); the coding region sequence and the full length of the *Aabrm1* (ID: 29120615) gene in *A. alternata* (Strain: SRC1lrK2f) were searched in the NCBI database. All primers required for the amplification of the target gene fragments are presented in Table S2. The gel recovery products are connected to target vectors and transferred to DH5α, and the next step is to screen and sequence positive clones.

The BLAST online analysis tool in the NCBI database was used to analyze the homology of amino acid sequences, download homologous protein sequences of *Aabrm1* protein in other fungi, and construct a phylogenetic tree in MEGA 7.0 (bootstrap = 1,000). The ORF Finder was used to predict the open reading frame (ORF), and the conserved domain of the *Aabrm1* gene was determined using conserved domain search software in the NCBI database.

### 2.3 Targeted gene knockout and complementation

The upper and lower homologous arms of the *Aabrm1* gene with a length of approximately 1,000 bp were downloaded from the NCBI database; the WT strain DNA was used as a template for amplification (*Aabrm1-*up and *Aabrm1-*down fragment); then, the replacement vector *Aabrm1-up-pCHPH-Aabrm1-down* was constructed using the homologous recombination method (Supplementary Figure S1); the attached vector was introduced into the PEG-mediated WT protoplasts. All transformants were screened using PDA media with hygromycin B resistance (0.04 g L^−1^), and polymerase chain reaction (PCR) and real-time quantitative reverse transcription PCR (qRT-PCR) were performed (Table S2 and Table S3); the required primers are presented in Table S1.

The *Aabrm1* gene is attached to the *N-pCNEO* vector, and the attached vector was introduced into the PEG-mediated Δ*Aabrm1* strain protoplasts. All transformants were screened using the PDA media with G-418 sulfate (0.25 g L^−1^), and PCR verification was performed (Table. S2); the required primers are presented in Table S1.

### 2.4 Phenotype analysis

#### 2.4.1 Melanin content determination

The extraction of *A. alternata* melanin followed the methods proposed by Zhang et al. (2021) and Gao et al. (2015) with a few modifications. After 5 days of incubation in a potato dextrose broth (PDB) medium, the WT and Δ*Aabrm1* and Δ*Aabrm1-C* hyphae are collected after filtering with four layers of sterile gauze, dried, and then ground into powder. The extracellular melanin was determined as follows: the filtrate was centrifuged for 10 min and adjusted to pH = 2 with 7 M HCl at 10,000 rpm for 15 min, and the supernatant was discarded to obtain the coarse extraction. The intracellular melanin analysis was as follows: 0.25 g of hyphae was dissolved in 1 M NaOH after boiling for 5 h in a water bath. After cooling, the filtrate was obtained using double-layer filter paper and adjusted to pH = 2 with 7 M HCl. Furthermore, centrifugation was performed at 10,000 rpm for 30 min, and the pellet was a crude extract of melanin. After three times of purification experiments, the pellet was dissolved and fixed in 1 M NaOH; the absorbance of the solutions was determined with a UV spectrophotometer at 400 nm.

#### 2.4.2 Radial growth assays and observation of morphology of spore, hyphae, and colony

The colony growth and sporulation followed the method by Li et al. (2023), WT, Δ*Aabrm1,* and Δ*Aabrm1-C* colony diameters were measured using the cross-method, and colony morphologies were recorded. Spores and hyphal morphologies of WT, Δ*Aabrm1,* and Δ*Aabrm1-C* strains were observed following the method reported by Li et al. (2022); the changes in the morphology of spore and hyphae were observed using scanning electron microscopy (JSM- 5600LV) and light microscopy.

### 2.5 Infection structural differentiation assays

According to the method reported by Huang et al. (2020), the GelBond membrane was cut to a 5 cm × 2 cm shape and placed on a clean glass slide, and 20 μL of spore suspensions of strains was added to the hydrophobic surface. The slides were moved into a Petri dish lined with moist filter paper on the bottom, placed in the dark at 28°C in a thermostatic incubator, and removed and count the spore germination and appressorium formation after 2, 4, 6, and 8 h, respectively. The 100 spores were counted each time, and 3 biological replicates were performed.

### 2.6 Penetration ability assays

The penetration ability was based on the method by Li et al. (2019). Mycelial plugs (5 mm × 5 mm) of WT, Δ*Aabrm1,* and Δ*Aabrm1-C* strains were added to PDA plates covered with sterile cellophane and cultured at 28°C for 2 days. Then, the cellophane was torn off, but the strains continued to culture for 3 days and the morphology was recorded.

### 2.7 Stress adaption assays

The stress resistance ability of WT, Δ*Aabrm1,* and Δ*Aabrm1-C* mycelia was investigated using the method by Wei et al. (2016) with slight modifications. The sodium dodecyl sulfate (SDS), Congo red (CR), H_2_O_2_, and menaquinone were added to PDA media, respectively. In total, 2 μL of spore suspensions was added to the PDA media and cultured for 5 days under darkness conditions; colony diameters were measured, and colony morphology was recorded. The stress resistance ability of spores followed the method by Kheder et al. (2012), with slight modifications. The spore suspensions were treated with ultraviolet (20 min) and H_2_O_2_ (3 mM). After 4 h of incubation, the spore germination rate was counted, 100 spores each time, and performed three times of biological replicates.

### 2.8 ROS production determination

#### 2.8.1 Fluorescence microscope observation

Intracellular ROS staining was performed with 2, 7-dichlorodihydro-ceindiacetate (DCFH-DA) in accordance with the method by Sun et al. (2021); DCFH-DA was prepared with DMSO reagent at 1 mg/mL concentration and stored at −20°C for future use. In total, 1 mL spore suspensions of WT, Δ*Aabrm1,* and Δ*Aabrm1-C* strains were prepared and placed in a 1.5 mL centrifuge tube, centrifuged for 10 min at 8000 xg at 4°C, and the supernatant was removed. The precipitates were suspended in 1 mL PBS (0.01 M, pH 7.2–7.4) buffer solution, and 10 μL of DCFH-DA dye solution was added. The solution was placed in an incubator at 37°C away from light and stained for 30 min, washed twice with 1 mL PBS buffer solution, and then suspended again. Finally, 20 μL spore suspensions were absorbed and observed under a fluorescence microscope and photographed.

#### 2.8.2 H_2_O_2_ and O_2_–content determination

The content of O_2_– was determined using the method of Wang et al. (2023). The oxygen-free radical (OFR) kit (Suzhou Keming Biotechnology Co., Ltd) was used to determine the O_2_^.-^ content. The 0.1 g liquid nitrogen ground hyphae of strains were collected and added to 1 mL extract for grinding under ice bath conditions, then centrifuged for 20 min, and the supernatant was placed on ice to be measured. The absorbance was measured at 550 nm; the corresponding value is found from the standard curve; the O_2_^.-^ content was expressed as nmol g^−1^. The content was measured by the H_2_O_2_ kit (Suzhou Keming Biotechnology Co., LtdS), the principle is that H_2_O_2_ and titanium sulfate can form a yellow titanium peroxide complex, and the product formed a characteristic absorption peak at 415 nm, finding the corresponding H_2_O_2_ content from the standard curve and expressed as μmol g^−1^.

### 2.9 Virulence assay

#### 2.9.1 Wound inoculation on pear fruit

The wound inoculation pathogenicity experiment used the method of Li et al. (2016), the pear fruits of uniform size and no mechanical injuries were selected and soaked in water containing 1% NaClO for 2 min. After the surface moisture was dried, a stainless-steel nail was used to create a wound (3 × 3 mm) along the equator of the fruits, and the wound sites were inoculated with 20 μL WT, Δ*Aabrm1,* and Δ*Aabrm1-C* strain spore suspensions. The diameter and morphology of disease spots were measured over a certain period of time. This experiment was measured using 15 pear fruits and was repeated three times.

#### 2.9.2 Non-wound inoculation on pear leaf

The non-wound inoculation pathogenicity experiment used the method proposed by Li et al. (2023). Pear leaves were washed with sterile water and dried, and the surface was inoculated with 10 μL WT, Δ*Aabrm1*, and Δ*Aabrm1-C* strain spore suspensions and cultured for 3 days, and then the disease spots were observed. This experiment was measured using 15 leaves and was repeated three times.

### 2.10 Quantitative RT-PCR analysis

The quantitative RT-PCR analysis system at the relative transcript level is shown in Table. S3. The *GAPDH* was used as a housekeeping gene, and the gene expression levels of melanin synthesis genes were by the 2^–ΔΔCt^ method (Liva and Schmittgen, 2001).

### 2.11 Statistical analysis

All charts and tables were drawn using Origin 8.0 and Microsoft Office 2010 and Microsoft Excel 2016 was used for data aggregation, standard deviation, and average. Duncan’s multiple run was measured using SPSS 18.0 (*p* < 0.05).

## 3 Results

### 3.1 Identification of *Aabrm1*

**Figure 1:**
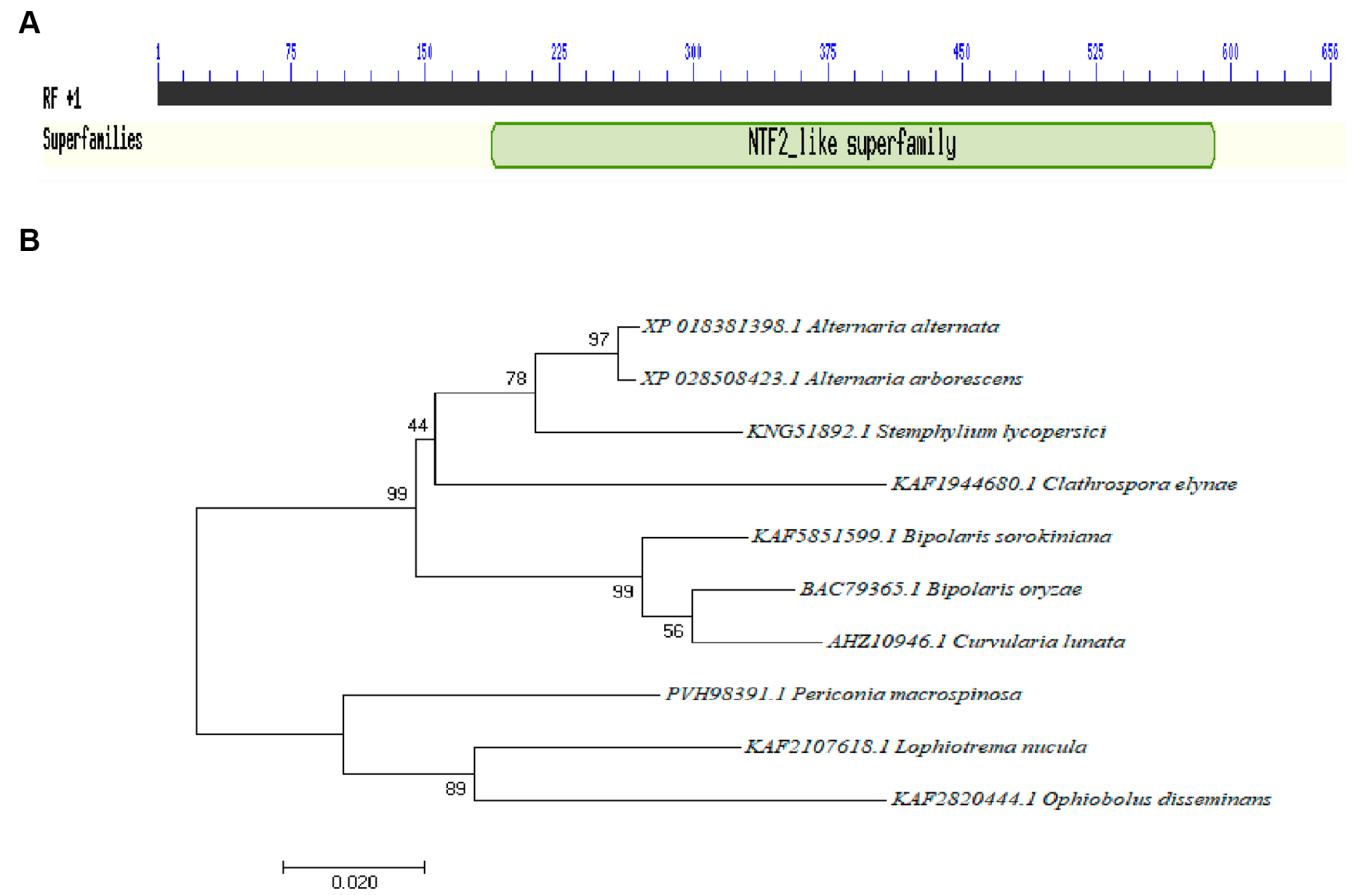
Identification of the *Aabrm1* gene conserved domains **(A)** and phylogenetic analysis **(B)** in *A. alternata.*

The total length of the *Aabrm1* gene is 654 bp, and its protein contains 185 amino acids; this sequence has three exons and two introns; the Aabrm1 protein-conserved domains indicated that it belonged to nuclear transport factor 2 (NTF2) family, and this family includes members of the NTF2 family, such as delta-5-3-ketosteroid isomerases, scytalone dehydratases, and the beta subunit of ring hydroxylating dioxygenases. The scytalone dehydratases can catalyze two reactions in the biosynthetic pathway that produces fungal melanin (Figure 1A). The phylogenetic tree was conducted by MEGA 7.0 software, and the *Aabrm1* has the highest affinity of *Alternaria arborescens* (Figure 1B), with sequence similarity as high as 99.46% (Results not shown).

### 3.2 Targeted deletion and complementation of *Aabrm1*

The PCR amplification yielded homologous arm products *Aabrm1*-up and *Aabrm1*-down with lengths of 1,129 bp and 971 bp (Supplementary Figure S2A). They were individually attached to *pCHPH*-linearized vectors to construct replacement vectors with a growth of approximately 4.4 kb (Supplementary Figure S2B). Transformants were obtained using a PEG-mediated protoplast transformation method, and no band appeared with cloned primers; the expression of the *Aabrm1* gene is low and close to 0, indicating that the Δ*Aabrm1* mutant strain was successfully constructed (Supplementary Figures S2C,D).

### 3.3 Deletion of *Aabrm1*-decreased melanin synthesis

**Figure 2:**
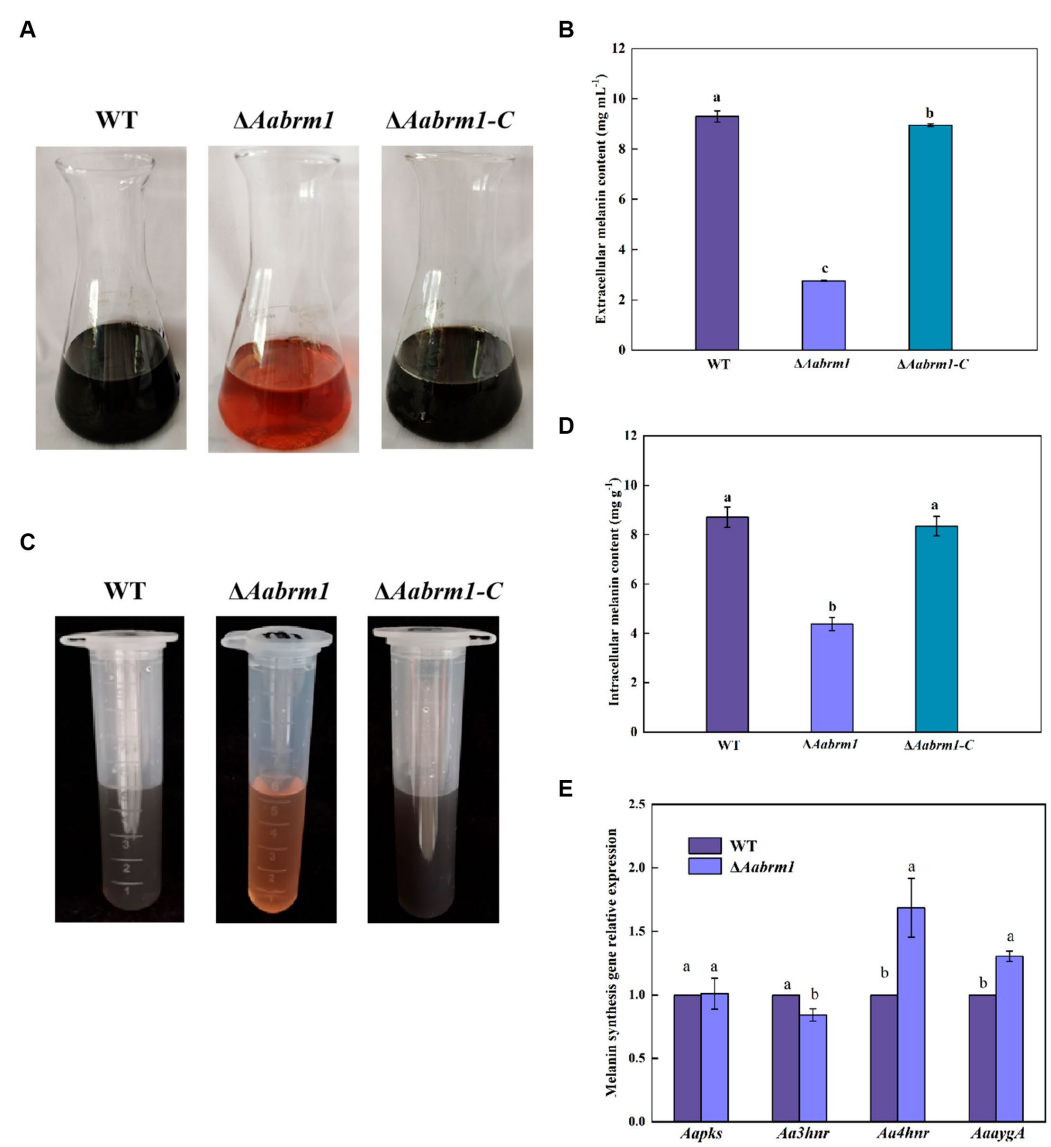
The effect of *Aabrm1* on melanin content of *A. alternata.* Extracellular melanin content of WT, Δ*Aabrm1,* and Δ*Aabrm1-C* strains **(A,B)**, intracellular melanin content of WT, Δ*Aabrm1,* and Δ*Aabrm1-C* strains **(C,D)**, and the relative expression level of melanin synthesis genes (*Aapks*, *Aa3hnr*, *Aa4hnr,* and *AaaygA*) in WT and Δ*Aabrm1* strains **(E)**. Lowercase letters indicate differences within the groups. Different capital letters indicate significant differences among different strains at *p* < 0.05.

To detect the effect of the *Aabrm1* gene on melanin synthesis, the contents of intracellular and extracellular melanin were determined by shaking WT, Δ*Aabrm1,* and Δ*Aabrm1*-C with PDB liquid medium for 5 days; the results showed that the extracellular filtrate of WT and the complementary strain were black, and Δ*Aabrm1* mutant strain was orange-red (Figure 2A); the extracellular melanin content of Δ*Aabrm1* strain is only 29.6% that of the WT (Figure 2B), and the content of intracellular melanin in the Δ*Aabrm1* strain was 50.29% of that of WT strain (Figures 2C,D). There was no significant change in the relative expression level of other melanin synthesis genes (*Aapks*, *Aa3hnr*, *Aa4hnr*, and *AaaygA*) in Δ*Aabrm1* strains (Figure 2E). The above results showed that the *Aabrm1* gene plays an important role in melanin synthesis.

### 3.4 *Aabrm1* gene is not necessary for vegetative growth But affects The morphology of spores and hyphae of *Alternaria alternata*

**Figure 3:**
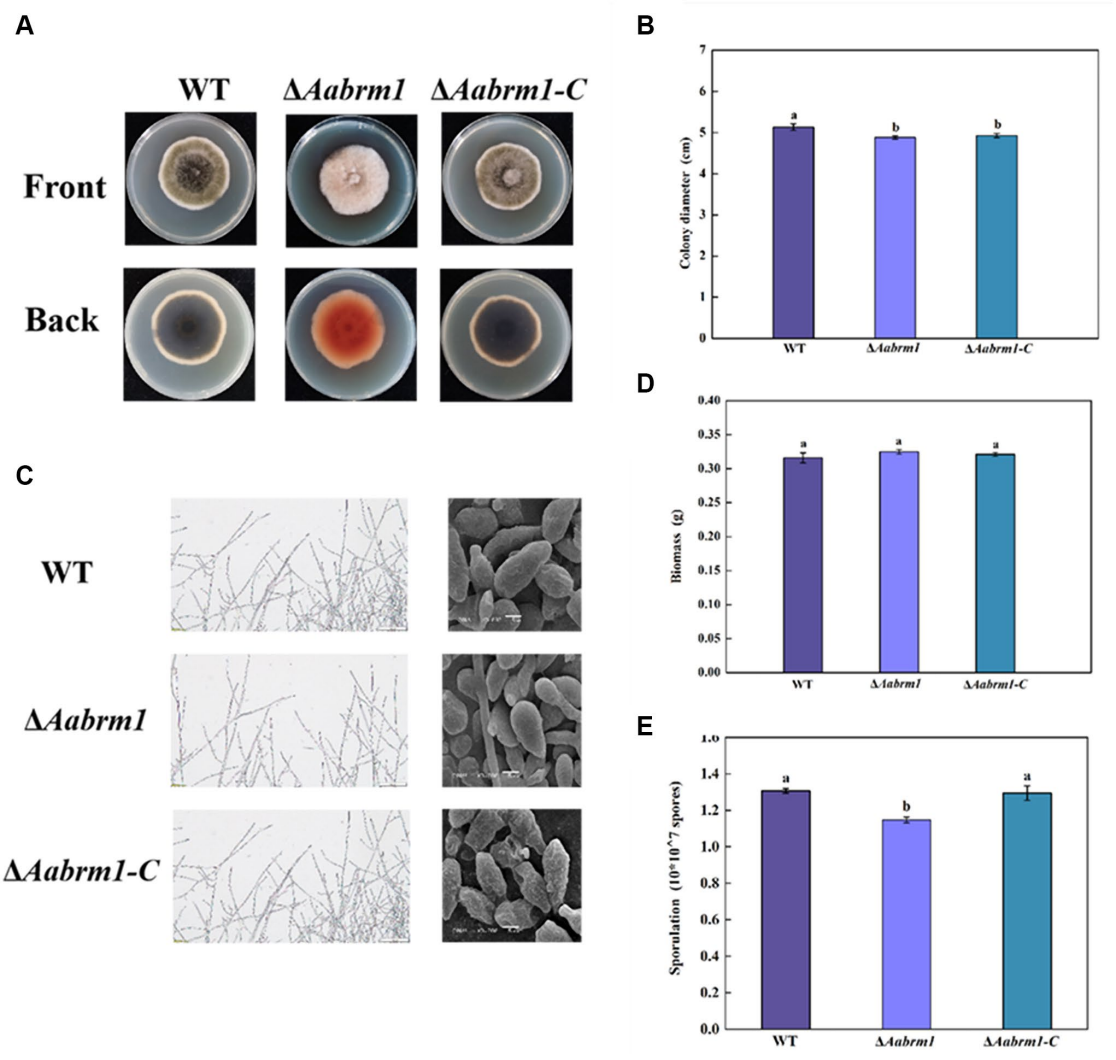
Effect of *Aabrm1* on colony morphology **(A)**, colony diameter **(B)**, mycelial and spore morphology **(C)**, biomass **(D)**, and sporulation **(E)** of *A. alternata.* The vertical line indicates standard error (±SE). Lowercase letters indicate differences within the groups. Different letters indicate significant differences (*p* < 0.05).

To assess the effect of the *Aabrm1* gene on growth and development, the morphology and colony diameter were recorded on PDA media for 3, 5, and 7 days, respectively. The colony color of Δ*Aabrm1* mutant became lighter, and a large amount of orange substance was produced on the back of the PDA medium (Figure 3A). The Δ*Aabrm1* colony diameter was slightly smaller than WT during the early incubation period, and there was no difference in the growth rate for 5 days (Figure 3B). Microscopic observations showed that aerial hyphae of WT and Δ*Aabrm1*-C strains grew more vigorously than Δ*Aabrm1* strain, the spore of Δ*Aabrm1* undergo a marked change in morphology, with smaller spore lengths and smoother spore surfaces (Figure 3C), and deletion of the *Aabrm1* gene had no effect on biomass accumulation (Figure 3D). The sporulation of Δ*Aabrm1* was significantly lower than WT (Figure 3E). These above results indicated that the *Aabrm1* gene did not have a significant effect on the growth of *A. alternata*, but it was involved in the normal development of spores and hyphae.

### 3.5 *Aabrm1* gene is crucial for *Alternaria alternata* infection at an early stage

**Figure 4:**
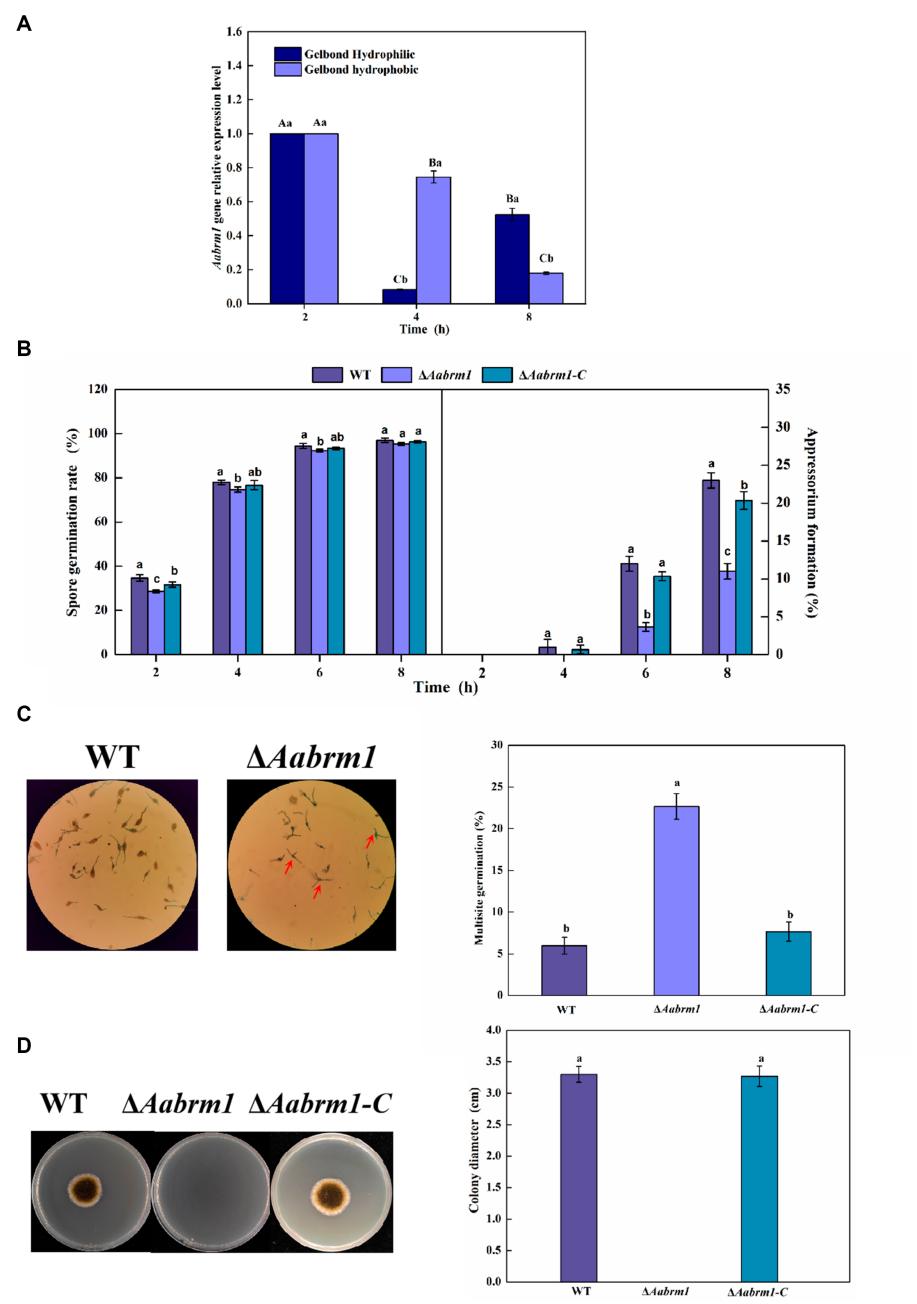
The *Aabrm1* gene relative expression level of spore germination (2 h), appressorium formation (4 h), and infection mycelium formation (8 h) on the GelBond hydrophobic and hydrophilic films **(A)**, effect of *Aabrm1* on spore germination and appressorium formation in GelBond membrane **(B)**, multiple germination **(C)**, and mycelia penetration **(D)** of *A. alternata*. The spores indicated by the red arrows represent multiple germination sites in a single spore. The vertical line indicates standard error (±SE). Lowercase letters indicate differences within the groups. Different letters indicate significant differences (*p* < 0.05).

To test the effect of the *Aabrm1* gene on the infection ability, the relative expression level was measured at infection structural differentiation stages of *A. alternata* on the GelBond hydrophobic and hydrophilic film and compared with appressorium formation (4 h) and infection mycelium formation (8 h), *Aabrm1* plays an important role in spore germination stage (2 h) (Figure 4A). The hydrophobic surface of the GelBond membrane was dropped with spore suspensions of WT, Δ*Aabrm1,* and Δ*Aabrm1-C,* to observe the infection structural differentiation. The spore germination rate of *ΔAabrm1* was lower than that of WT and Δ*Aabrm1-C* at 2, 4, and 6 h. At 8 h, there was no difference between the WT and Δ*Aabrm1-C* strains. All strains did not produce appressorium at 2 h, and the appressorium formation rate of Δ*Aabrm1* was lower than that of WT and Δ*Aabrm1-C* strains at 4, 6, and 8 h (Figure 4B). An interesting phenomenon was found during the spore germination of Δ*Aabrm1* strain, many spores produced multiple germination tubes, and there were more than the WT and Δ*Aabrm1-C* strains (Figure 4C). In addition, the hyphae penetration ability showed that the Δ*Aabrm1* strain failed to penetrate cellophane to form colonies, but the WT and Δ*Aabrm1-C* strains successfully penetrated and formed colonies (Figure 4D). The above results revealed that the *Aabrm1* gene plays an important role in infection structure formation and penetration of *A. alternata*.

### 3.6 *Aabrm1* gene contributes to stress resistance of *Alternaria alternata*

**Figure 5:**
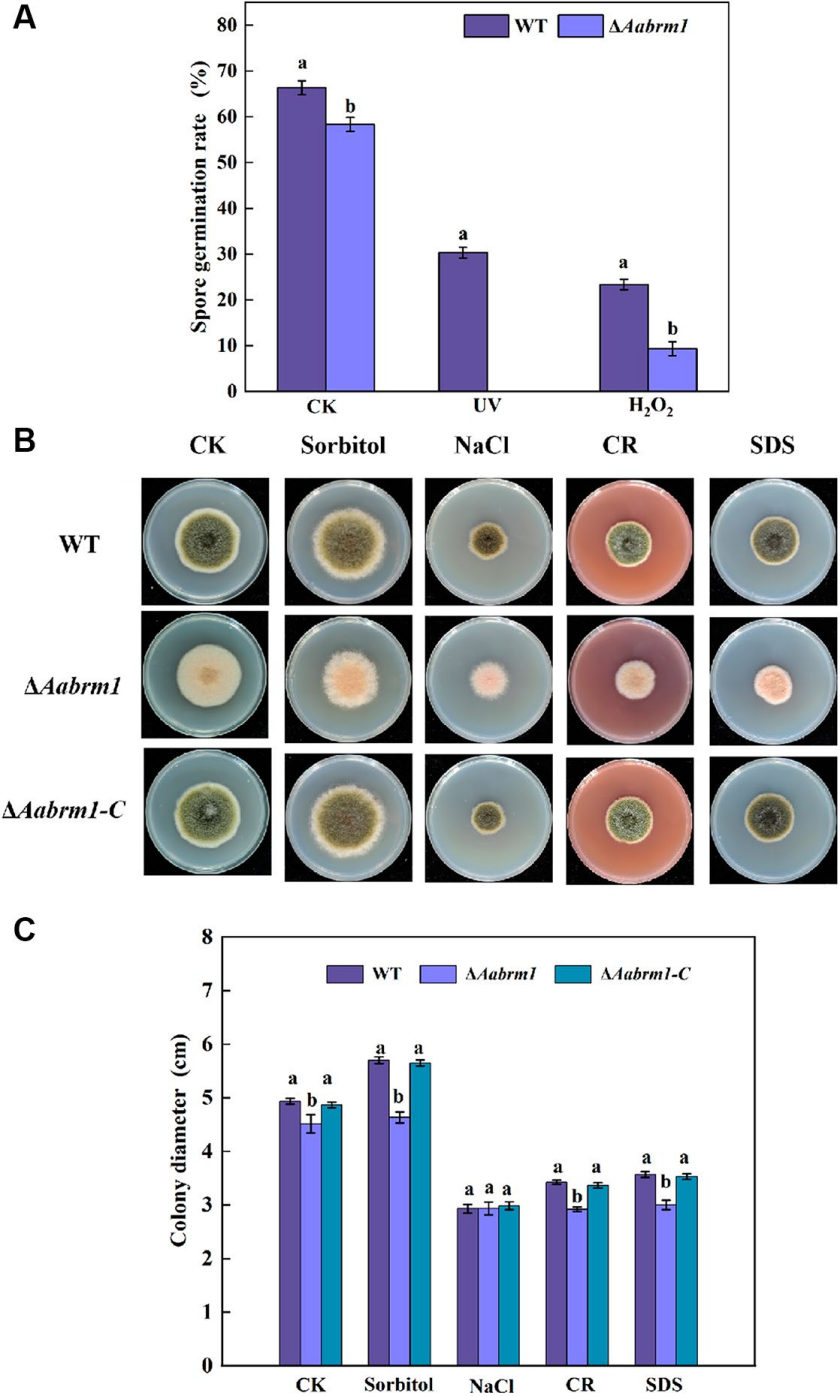
Effect of the WT, Δ*Aabrm1,* and Δ*Aabrm1-C* strains on the ultraviolet and H_2_O_2_ stress resistance ability of spores **(A)**, the mycelia stress resistance ability of WT, Δ*Aabrm1,* and Δ*Aabrm1-C* strains grown on PDA media supplemented with Sorbitol, NaCl, Congo red, and SDS **(B,C)**. Lowercase letters indicate differences within the groups. Different capital letters indicate significant differences among different strains at *p* < 0.05.

When fungal spores lack melanin, the ability of spores to cope with adverse external environments is reduced. Under ultraviolet stress, the Δ*Aabrm1* mutant spores did not germinate, and under H_2_O_2_ stress, the spore germination rate of Δ*Aabrm1* mutant strain was 39.91% that of the WT; the Δ*Aabrm1* is more sensitive to ultraviolet stress (Figure 5A). Under osmotic stress, after treatment with 1 M sorbitol, the growth inhibition rates of WT- and Δ*Aabrm1-*mutant mycelia were − 13.51 and − 2.38%, and the inhibitory rates of 1 M NaCl after treatment were 68.26 and 54.27%, respectively. The Δ*Aabrm1* mutant strains were significantly more sensitive to cell wall synthesis disruptors than WT and Δ*Aabrm1-C* strains, with inhibitory rates of 54.79 and 50.67% after CR and SDS treatment, respectively (Figures 5B,C). The above results showed that the *Aabrm1* gene mediates the synthesis of melanin and plays an important role in the stress resistance of *A. alternata*.

### 3.7 Deletion of *Aabrm1* attenuates the ability to cope with ROS of *Alternaria alternata*

**Figure 6:**
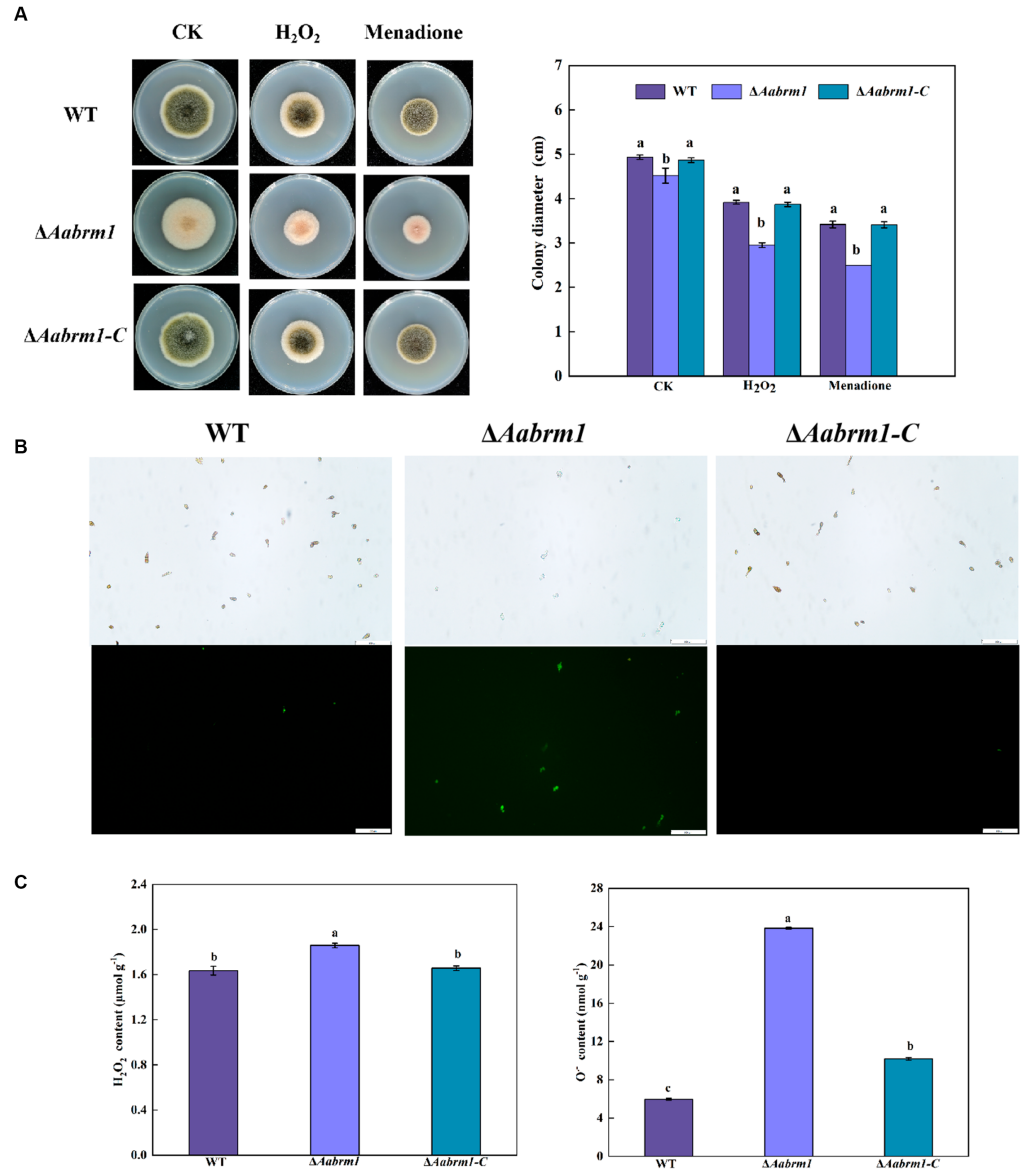
Effect of the WT, Δ*Aabrm1,* and Δ*Aabrm1-C* strains grown on PDA media supplemented with H_2_O_2_ and menaquinone **(A)**, intracellular ROS staining (bar scale 100 μm) **(B)**, and H_2_O_2_ and O_2_–contents **(C)**. Lowercase letters indicate differences within the groups. Different capital letters indicate significant differences among different strains at *p* < 0.05.

The PDA media supplemented with H_2_O_2_ and menaquinone produced a more pronounced inhibitory effect on Δ*Aabrm1* mutant strain, its growth inhibition rates were 53.22 and 80.8%, and the growth inhibition rates of the WT strain were 25.77 and 44.15%, respectively (Figure 6A). The green fluorescence intensity of DCFH-DA staining is proportional to the level of intracellular ROS. The staining results revealed that the fluorescence intensity of WT and Δ*Aabrm1-C* strains was faint and scarce; however, the Δ*Aabrm1* spores were starry and numerous (Figure 6B). Further assays showed that the contents of H_2_O_2_ and O_2_^.-^ in the Δ*Aabrm1* mutant strain were higher than those in the WT and Δ*Aabrm1-C* strains; the content of O_2_^.-^ in the Δ*Aabrm1* mutant strain was as high as 3.98 times that of the WT (Figure 6C). The above results clarified that the *Aabrm1* gene mediates the synthesis of melanin and plays an important role in *A. alternata* in response to oxidative stress.

### 3.8 *Aabrm1* gene is involved in the virulence of *Alternaria alternata*

**Figure 7:**
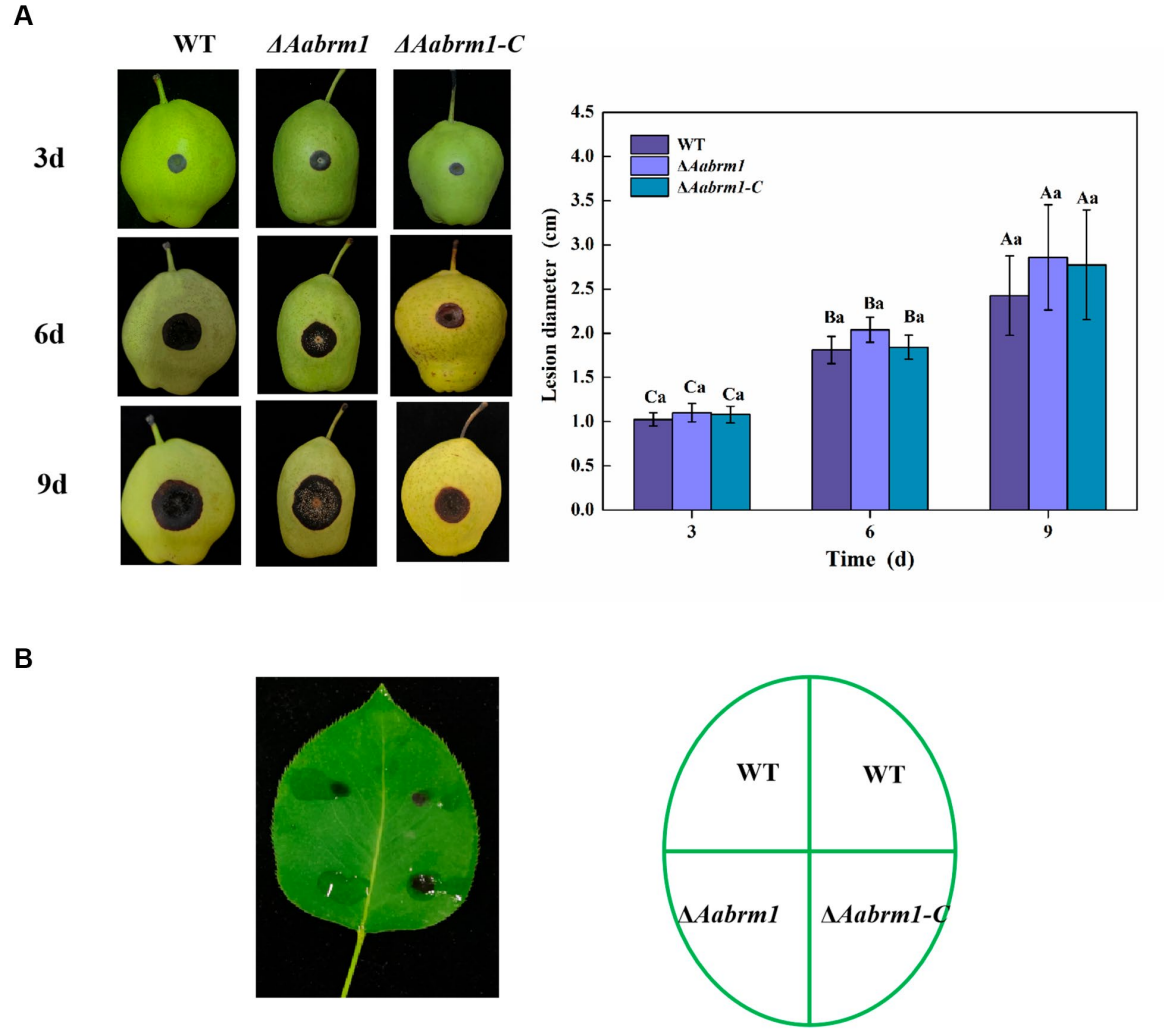
Effect of *Aabrm1* on pathogenicity was observed on wound-inoculated pear fruits **(A)** and non-wound-inoculated pear leaves **(B)**. Vertical lines indicate standard error (±SE) of the means. Uppercase letters indicate differences between the groups. Lowercase letters indicate differences within the groups. Different letters indicate significant differences (*p* < 0.05).

To evaluate the effect of the *Aabrm1* gene on the pathogenicity of *A. alternata*, the development of black spots was observed on wound-inoculated pear fruit and non-wounded inoculated pear leaves. Compared with WT and Δ*Aabrm1-C* strains, there was no significant difference in lesion diameter of Δ*Aabrm1* on pear fruit (Figure 7A). However, Δ*Aabrm1* did not form obvious spots like WT and Δ*Aabrm1-C* strains on pear leaves (Figure 7B). These results further confirmed that *Aabrm1* is involved in early infection of *A. alternata.*

## 4 Discussion

In many plant pathogenic fungi, melanin and its biosynthesis have been widely studied as important secondary metabolites (Tsuji et al., 2000; Kihara et al., 2008; Fetzner et al., 2014; Wang Y. et al., 2018). Genes involved in the melanin synthesis pathway in *A. alternata* have been reported, including melanin synthesis transcription factor *AacmrA*, polyketide synthase gene *Aapks*, reductase gene *Aahnr*, a-hydrolase gene *Aaayg*, scytalone dehydratase gene *Aabrm1*, and laccase gene family *Aalac1-7* (Gao et al., 2022). Although *Aabrm1* in *A. alternata* had been reported, but only focused on perylene quinone biosynthesis, other biological functions still need to be further revealed. In this study, *Aabrm1* was cloned in *A. alternata*, the causal agent of pear black spot, which is 654 bp in length, contains a conserved Scytalone_dh domain (Figure 1) and has been consistently reported in many fungi (Hao et al., 2022; Xue et al., 2022).

In this study, the contents of both intracellular and extracellular melanin in the Δ*Aabrm1* mutant were reduced (Figure 2), and the deletion of the *Aabrm1* gene in the PDA medium had little effect on colony diameter but significantly changed colony color. Light brown mycelium was formed on the front side of the medium, and many orange substances were formed on the back side (Figure 3A), which was consistent with the results of *Colletotrichum gloeosporioides* (Wang et al., 2020). In addition, the spore morphology of *ΔAabrm1* mutant was smooth (Figure 3C), which was consistent with the results of the tomato pathotype melanin-deficient strain of *A. alternata* (Kheder et al., 2012). Moreover, our previous study also showed that the lack of melanin causes the surface of the spores to become smooth in *A. alternata* (Li et al., 2022, 2023). As an important secondary metabolic substance, melanin is involved in the normal growth and development of fungal spores (Cordero and Casadevall, 2017); the cell wall integrity of fungus was also changed after the absence of melanin (Li et al., 2023).

Infection structures including infection cushion, appressorium, penetration peg and haustorium formed by specialized hyphae are essential for plant pathogenic fungi invading and establishing colonization relationships with host tissue (Goyet et al., 2017), and melanin usually accumulates in the appressorium, resulting in high turgor pressure, the mechanical driving force formed by this swelling pressure causes the penetration peg to enter the host (Wang et al., 2005). However, *A. alternata* forms the colorless appressorium without melanin accumulation. Interestingly, the infection structure formation of the Δ*Aabrm1* strain was significantly delayed, and its penetration ability was significantly reduced (Figure 4); similar research studies have also been reported in *V. dahliae*, which also produces colorless infection structure (Luo et al., 2016), indicating that *Aabrm1* or its mediated melanin synthesis may be involved infection structure differentiation of fungi. Research has also found that both melanin synthesis and infection of cotton by *V. dahliae* are coupled through transmembrane protein *VdSho1*, and the MAPK signal module Ste50-Ste11-Ste7 is involved in this coupling process (Li et al., 2019). In *A. alternata*, sho-MAPK, cAMP, and Ca^2+^ signaling pathways are involved in infection structure formation induced by physicochemical cues from pear fruit peel wax, affecting the synthesis of melanin at the same time (Liu et al., 2021; Zhang et al., 2021; Jiang et al., 2023). The above reports suggest that melanin may be directly or indirectly involved in infection structure formation or infection process in plant pathogenic fungi, which only form colorless appressorium. However, its detailed molecular regulatory mechanism needs to be further elucidated.

Melanin is beneficial to organisms to resist various adverse environmental stresses, such as ultraviolet radiation, hypertonic stress, extreme temperature, and heavy metal stress, hence it is called “fungal armor” (Toledo et al., 2017; Jia et al., 2021). The data presented in this study indicated that *Aabrm1-*mediated melanin synthesis is beneficial to *A. alternata* resistance; the spores of Δ*Aabrm1* are more sensitive to both high temperature and ultraviolet stress (Figure 5A), which is consistent with the previous research results of tomato pathotype *A. alternata* (Kheder et al., 2012). The Δ*Aabrm1* strain is sensitive to sorbitol and cell wall integrity inhibitor and is more sensitive to oxidative stress (Figures 5, 6), which is consistent with the results of our previously constructed melanin synthesis-deficient strain (Li et al., 2022, 2023), and the similar phenomena have also occurred in *C. gloeosporioides* (Wang et al., 2021). Fungi have a complex ROS production and scavenging system for the maintenance of cell oxidation–reduction balance. A large amount of O_2_^.-^ and H_2_O_2_ accumulated in the Δ*Aabrm1* strain (Figures 6B,C). In *Coniothyrium minitans*, the deletion of melanin synthesis transcription factor *CmMR1* leads to a decrease in melanin and an increase in ROS accumulation (Luo et al., 2018). Oxidative stress is caused by an imbalance between ROS production and ROS clearance; melanin has the ability to scavenge reactive oxygen species (Helmut, 2015). Melanin is a powerful free radical quencher with strong antioxidant capacity (Cordero and Casadevall, 2017), and it is also able to maintain the cell stability of the fungus at extreme temperatures, possibly due to the presence of melanin in the cell wall, which reduces the pore size and has stronger resistance (Casadevall et al., 2017).

The role of DHN melanin in the pathogenic process of plant pathogenic fungi is diverse and dependent on fungal species. When melanin deposition occurs in the appressorium, the pathogenicity significantly decreases when melanin production is affected (He et al., 2017; Steiner and Oerke, 2017). Studies on some fungi have also shown that pathogenicity is not related to the formation of melanin (Liang et al., 2018; Derbyshire et al., 2019). In *Botrytis cinerea* and *Alternaria brassicicola,* there is a negative correlation between melanin and pathogenicity (Cho et al., 2012; Zhang et al., 2015). However, melanin also regulates the pathogenicity of some fungi with colorless infection structures, such as *V. dahliae* (Luo et al., 2016). Data presented in this study showed that the virulence of the Δ*Aabrm1* strain was reduced on non-wound-inoculated pear leaves but not changed in wound-inoculated pear fruits (Figure 7). These results further suggested that *Aabrm1* might be involved in early infection of *A. alternata.* However, its detailed molecular regulatory mechanisms need to be further elucidated.

## 5 Conclusion

In conclusion, melanin, as a secondary metabolic substance, is essential for the development and pathogenicity of *A. alternata*. The *Aabrm1*-mediated melanin synthesis plays an important role in the growth and development and oxidative stress of *A. alternata*. Interestingly, in the present study, data showed that *Aabrm1* was involved in the early infection of *A. alternata* with colorless appressorium formation through affecting infection structure formation induced by pear wax extract and infecting process after inoculating non-wounded pear leaves. These findings provide a new insight into *Aabrm1*-mediated melanin biosynthesis on the pathogenicity of *A. alternata.*

## Data availability statement

The raw data supporting the conclusions of this article will be made available by the authors, without undue reservation.

## Author contributions

RL: Writing – original draft, Writing – review & editing. YL: Funding acquisition, Project administration, Supervision, Writing – review & editing. WX: Formal analysis, Visualization, Writing – original draft. WL: Data curation, Writing – original draft. XX: Software, Writing – original draft. YB: Methodology, Resources, Writing – review & editing. DP: Conceptualization, Writing – review & editing.
