# Supplementary material for: Aabrm1-mediated melanin synthesis is essential to growth and development, stress adaption, and pathogenicity in Alternaria alternata
Source: Front Microbiol. 2024 Jan 11;14:1327765. doi: 10.3389/fmicb.2023.1327765 (PMC10808324; doi:10.3389/fmicb.2023.1327765)
Supplement: Supplementary file 1 [file Data_Sheet_1.pdf]

Supplementary Table 1

Primers used in this study

Gene	Primer sequences (5' - 3')
Aabrm1-F	ATGTTTGAGA AGAACGAATT
Aabrm1-R	CTACAACGCC TGACCAGT
Aabrm1-up-F	ACAGCTATGACCATGATTACGAATTCCGTCTTCGAGCGTCTTTCCT
Aabrm1-up-R	GATCCCCGGGTACCGAGCTCGAATTCTCCTGCGCCATATTAGCATT
Aabrm1-down-F	CATGCATGGTTGCCTAACTCGGCGCGCCGCTTATGTGCGGATAGAGTTG
Aabrm1-down-R	GACGGCCAGTGCCAAGCTTCGGCGCGCCTCCCGATTTCTTCACGATGT
Aabrm1-Compfor	GCATGGACGAGCTGTACAAGGAGCTC ATGTTTGAGAAGAACGAATT
Aabrm1-Comprev	ATGGAGCTATTAAATCACTATCTAGA CTACAACGCCTGACCAGT
Aabrm1-qPCR-F	GCCCACAGTTTCAACATGCA
Aabrm1-qPCR-R	CCACCTGATATCCGGGTTCA
Aapks-qPCR-F	TGGTAACACCGCGGTCATC
Aapks-qPCR-R	GCTCACTGTCACCATGTGCAT
Aabrm2-qPCR-F	CTTGACCGTGGAAGGAAATCA
Aabrm2-qPCR-R	TTGGGTGGAGGATCGAAGTG
Aabrn2-qPCR-F	ACAGTTCTTCGTCGGCCAAA
Aabrn2-qPCR-R	CAGCTGCGATGGAGCTCAT
AaaygA-qPCR-F	CACATCAACATCGCAACC
AaaygA-qPCR-R	GCGGGACAGTCTCCAGTA
GAPDH-F	CTTACTGCCTCCACCAACTG
GAPDH-R	TGACGTTGGAAGGAGCGAAG

Aabrm1 (XM_018535021.1), *Aapks* (XM_018528048.1), *Aabrm2*(XM_018532026.1), *Aabrn2*(XP_018383345.1), *AaaygA*(XM_018525364.1)

Supplementary Table 2

PCR reaction system for the target gene fragment

Name	Dosage (μL)
2×SYBR® Green Pro Taq HS Premix	10
forward primer (10 μM)	0.5
reverse primer (10 μM)	0.5
cDNA	1
ddH_2O	8

Supplementary Table 3

Quantitative RT-PCR analysis for gene expression

Name	Dosage (μL)
2×SYBR® Green Pro Taq HS Premix	10
forward primer (10 μM)	1
reverse primer (10 μM)	1
cDNA	1.5
ddH_2O	6.5

Supplementary Figure 1

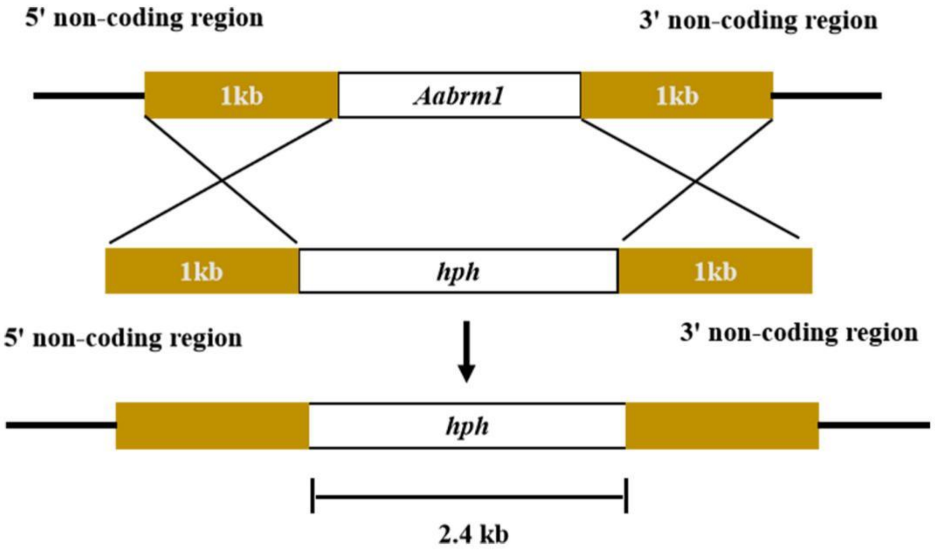

Homologous recombination method

Supplementary Figure 2

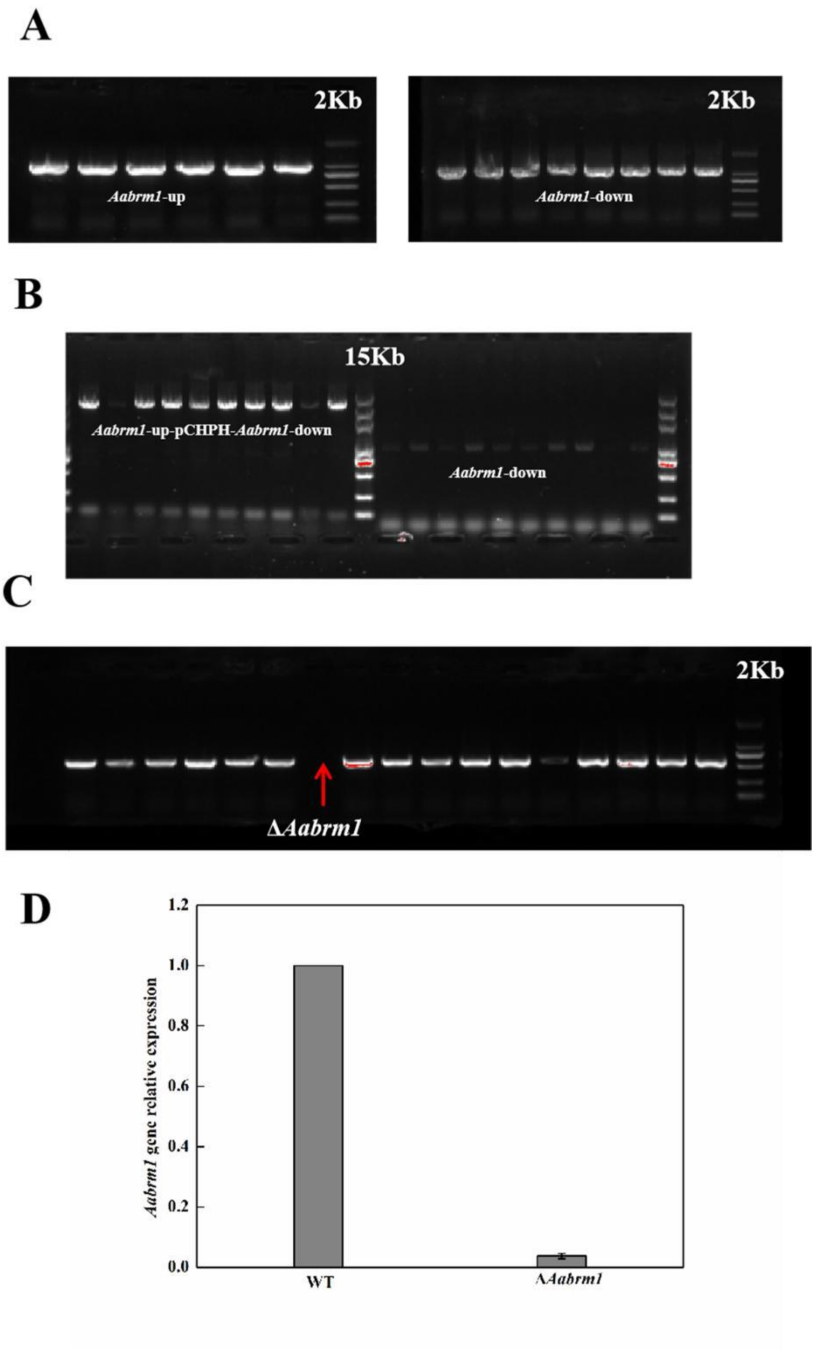

The PCR amplification yielded homologous arm products *Aabrm1*-up and *Aabrm1*-down(A), the *pCHPH* vector containing hygromycin-resistance gene (*HYG*) was connected with upstream and downstream target gene fragments (B), PCR verification of Δ*Aabrm1* strain (C) and the *Aabrm1* gene expression level of WT and *ΔAabrm1* (D).
